# Endothelial dysfunction in chronic obstructive pulmonary disease: an update on mechanisms, assessment tools and treatment strategies

**DOI:** 10.3389/fmed.2025.1550716

**Published:** 2025-02-12

**Authors:** Giuseppina Marcuccio, Claudio Candia, Mauro Maniscalco, Pasquale Ambrosino

**Affiliations:** ^1^Istituti Clinici Scientifici Maugeri IRCCS, Pulmonary Rehabilitation Unit of Telese Terme, Telese Terme, Italy; ^2^Department of Biomedicine, Neuroscience and Advanced Diagnostics, University of Palermo, Palermo, Italy; ^3^Department of Clinical Medicine and Surgery, University of Naples “Federico II, ” Naples, Italy; ^4^Istituti Clinici Scientifici Maugeri IRCCS, Scientific Directorate of Telese Terme Institute, Telese Terme, Italy

**Keywords:** COPD, endothelial function, cardiovascular risk, disability, exercise, rehabilitation

## Abstract

Chronic obstructive pulmonary disease (COPD) is a complex condition marked by chronic respiratory symptoms, such as cough and dyspnoea, and persistent irreversible airway obstruction, punctuated by acute episodes of exacerbations. COPD is associated with a significant mortality risk and several comorbidities, including cardiovascular diseases. The link between COPD, acute exacerbations and cardiovascular diseases has been recently acknowledged under the unifying concept of cardiopulmonary risk. In this context, endothelial dysfunction (ED) has been identified as a key contributor to the systemic manifestations of COPD and an early event in atherogenesis, thus potentially linking respiratory diseases and cardiovascular risk. Assessing endothelial dysfunction could therefore provide valuable prognostic insights into COPD, while targeting it may emerge as a promising therapeutic approach. Nonetheless, several aspects such as clinical assessment options and potential treatment strategies are still under debate, despite an intense research activity in recent years and promising results coming from the field of pulmonary rehabilitation medicine, which seems to be highly beneficial for the improvement of ED in COPD patients. On these premises, this mini review aims to provide an updated overview of the pathophysiology of ED in the context of COPD, with a focus on its assessment and its potential as an attractive therapeutic target.

## Introduction

Chronic obstructive pulmonary disease (COPD) is a complex condition marked by chronic respiratory symptoms, such as cough and dyspnoea, and persistent irreversible airway obstruction (1). Data from 2020 suggest that COPD affects ~10.6% of the global population (480 million people), with its prevalence projected to rise to 23% by 2050 (2). COPD is also the third leading cause of global mortality, heavily impacting quality of life, healthcare costs, and rehabilitation demands (1). Additionally, COPD is associated with several comorbid conditions, including cardiovascular diseases (CVDs), psychiatric disorders, and metabolic diseases (3, 4). Moreover, acute exacerbations of COPD (AECOPD) further complicate its course (5) and contribute to a faster worsening of lung function and to an increase in cardiovascular mortality (6). In this setting, the unifying concept of COPD-related cardiopulmonary risk, defined as the increased chance for a COPD patient to experience both major acute cardiovascular and respiratory events, was recently introduced to underscore the intimate link between COPD and CVDs (7).

While smoking is a known common risk factor between COPD and CVD, scientific evidence suggests that inflammation and oxidative stress may be crucial pathogenetic mechanisms in COPD patients, acting as key factors of disease progression (8). However, oxidative stress and chronic inflammation are not confined to the airways, but are more often systemic, thus affecting vascular integrity (9). Thus, also in light of research on convalescent coronavirus disease 2019 (COVID-19) patients (10), endothelial dysfunction (ED) has been recently recognized as a crucial factor for the systemic manifestations of several respiratory conditions, including COPD, as well as a key event in the process of atherogenesis, thus linking respiratory diseases and cardiovascular risk (8). However, the exact mechanisms underlying ED, along with clinical assessment options and potential treatment strategies for ED in COPD patients, remain subjects of debate despite significant research efforts in recent years. Moreover, no consensus has been reached regarding the most suitable assessment method or the treatment strategy with the greatest potential to improve ED.

In light of the above, this narrative mini review will offer a brief but comprehensive overview on the crucial mechanisms, the assessment tools and the therapeutical options currently available for ED among COPD patients, with the aim of providing updated evidence and practical advice on the topic.

## Endothelial dysfunction in COPD: mechanisms and mediators

**Figure 1:**
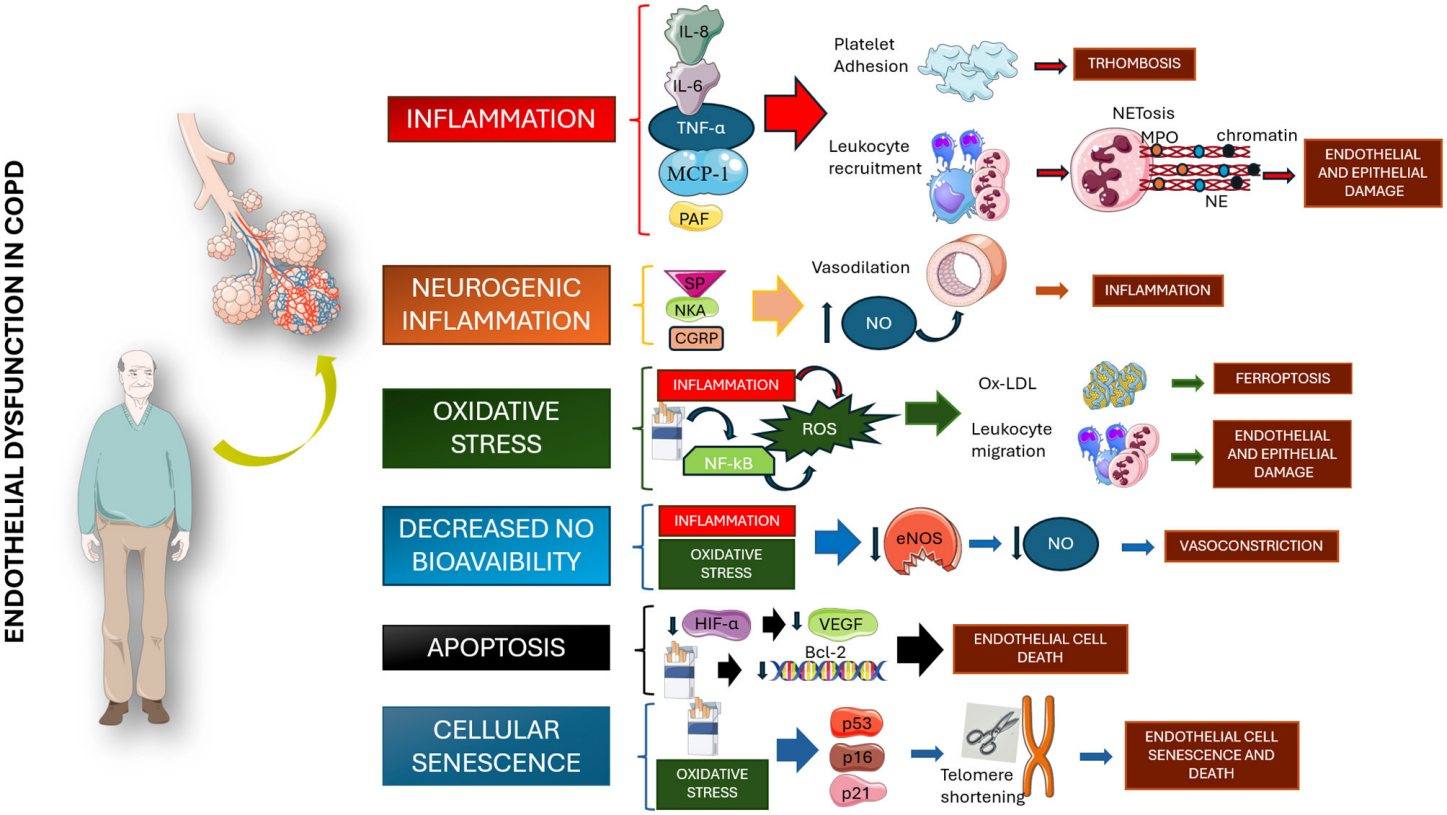
Pathogenetic mechanisms involved in the genesis of endothelial dysfunction among chronic obstructive pulmonary disease patients. All proposed mechanisms seem to be related to chronic inflammation, alteration in NO production, and cellular senescence. IL, interleukin; TNF-a, tumor necrosis factor a; MCP-1, monocyte chemoattractant protein; PAF, platelet-activating factor; NET, neutrophil extracellular traps; NE, neutrophil elastase; SP, substance P; NKA, neurokinin A; GCRP, calcitonin gene-related peptide; NO, nitric oxide; NF-kB, nuclear factor kappa-light-chain-enhancer of activated B cells; ROS, reactive oxygen species; Ox-LDL, oxidized low-density lipoprotein; eNOS, endothelial nitric oxide synthase; HIF-a, hypoxia-inducible factor 1-alpha; VEGF, vascular endothelial growth factor; Bcl-2, B cells lymphoma 2; p53, protein 53; p16, protein 16; p21, protein 21. Parts of Figure 1 were created using images from Servier Medical Art, licensed under CC BY 4.0.

ED involves vascular damage driven by systemic inflammation, hypoxia, and extracellular matrix injury through different mechanisms involving multiple pathways, some of them not yet fully understood, as shown in Figure 1 (11). As a result of these multiple interactions, endothelial cells (ECs) are structurally compromised and are impaired in their crucial physiological activities (12).

### Chronic and neurogenic inflammation

Chronic inflammation in COPD can disrupt intercellular adhesion molecules (ICAM) expression and function, facilitating increased leukocyte migration across the endothelium. Accordingly, elevated ICAM-1 levels have been correlated with respiratory decline and emphysema severity (13). Similarly, inflammatory mediators like interleukin (IL)-8, tumor necrosis factor (TNF)-α, and monocyte chemoattractant protein (MCP)-1 persistently recruit leukocytes, exacerbating COPD during acute exacerbations (14, 15). Neutrophil extracellular traps (NETs) also cause EC cytotoxicity and airflow limitation (16). Moreover, cigarette smoke worsens ED with several mechanisms, including an increased alveolar-capillary permeability and the creation of neo-epitopes, which elicit autoimmune responses (17). Particularly, carbonyl-modified antibodies and citrullinated proteins were found in higher concentrations in COPD patients-derived airway samples (18, 19).

Chronic inflammation also disrupts the function, mobilization, and survival of circulating stem cells, such as endothelial progenitor cells (EPCs) (20). These cells are typically recruited to sites of injury, where they differentiate into mature ECs and integrate into the damaged vasculature, aiding in the restoration of vascular integrity. However, this process appears to be impaired in COPD patients, potentially contributing to the development of ED and its associated biological and clinical consequences.

### Oxidative stress and NO bioavailability

Oxidative stress, a key driver of COPD progression and ED, arises from elevated reactive oxygen species (ROS) in plasma and ECs (21). ROS cause lipid peroxidation, activate the receptors for advanced glycation (RAGE), and induce ferroptosis. Additionally, oxidative stress reduces nitric oxide (NO) bioavailability, inducing accumulation of asymmetric dimethylarginine (ADMA) and prompting an increased arginase activity (9), thus impairing vasodilation (22). Finally, neopterin levels, a biomarker of chronic heart failure and systemic inflammation, have been shown to reflect immune responses during oxidative stress in COPD patients and correlate with reduced respiratory function (11, 23).

### Cellular senescence and apoptosis

Cellular senescence in ECs results from telomere shortening, mostly due to oxidative stress, and promoting chronic inflammation and EC apoptosis (24, 25). In COPD, cigarette smoke accelerates EC senescence and reduces anti-apoptotic agents like prostacyclin (PGI2), further impairing endothelial health (26).

## Assessment of endothelial function in COPD

Endothelial function can be evaluated using various techniques with differing levels of invasiveness. Here, we highlight the most effective methods for assessing ED in COPD patients.

### Laboratory biomarkers

Biomarkers for ED, including acute-phase proteins, cytokines, and adhesion molecules, can offer insights into the pathogenesis of COPD and its related cardiovascular risk (27). Elevated C-reactive protein (CRP) and fibrinogen levels have been linked to a reduced NO synthesis and increased coronary artery disease (CAD) risk (28, 29). Platelet activity markers, like P-selectin and mean platelet volume (MPV), also highlight a pro-thrombotic state in COPD patients (30, 31), while other generical markers of oxidative stress, such as peroxynitrite, ADMA, vascular cell adhesion molecule-1 (VCAM-1), and malondialdehyde have been used to assess ED, alongside circulating endothelial cells (CECs), EPCs, or endothelin-1 (ET-1), which are deemed to be more specific to endothelial function (32). In particular, studies have shown that EPC functionality declines in COPD due to apoptotic triggers (33). Microparticles, especially endothelial-derived microparticles (EMPs), are elevated in COPD and have been shown to correlate with reduced respiratory function and the severity of emphysema (34, 35). Finally, endocan, a proteoglycan protein and sensitive marker of ED, shows potential as a predictor of exacerbations (36). Nonetheless, some crucial issues still affect the current use of laboratory biomarkers in clinical practice, despite promising results from trials. To date no single biomarker has been demonstrated to hold capability of either diagnosing, predicting and/or stratifying the severity of ED with acceptable sensibility and specificity, at least in the clinical setting of COPD patients. Finally, the limited availability and high costs of laboratory assays for measuring cytokines, adhesion molecules, and certain cell populations may pose another barrier to the widespread implementation of laboratory biomarkers as tools for assessing ED in COPD patients.

### Clinical assessment strategies

Over the past 35 years, a variety of methodological approaches have been developed to study endothelial function in humans (37). Despite the widespread use of non-invasive methods for assessing endothelial function, which are valuable for the primary and secondary prevention of cardiovascular events, none of these methods are currently recommended for routine clinical practice according to existing guidelines (38).

The most commonly used techniques for evaluating endothelial function in research focus on post-occlusive reactive hyperemia One of the earliest methods, introduced in the early 20th century, is Venous Occlusion Plethysmography (VOP). This technique assesses the functionality of the venous system in the limbs, particularly in conditions such as chronic venous diseases, claudication, and diabetes mellitus (39–41). In brief, changes in the blood volume in the limbs are detected, thus indirectly measuring the local post-ischemic vascular tone (42).

Laser Doppler Flowmetry (LDF) measures endothelium-dependent dilation in small cutaneous vessels by applying the Fizeau-Doppler principle, which derives blood flow velocity from the frequency shifts in backscattered light caused by moving red blood cells (43). However, its reproducibility is limited due to the heterogeneity of the cutaneous microvasculature (44).

**Figure 2:**
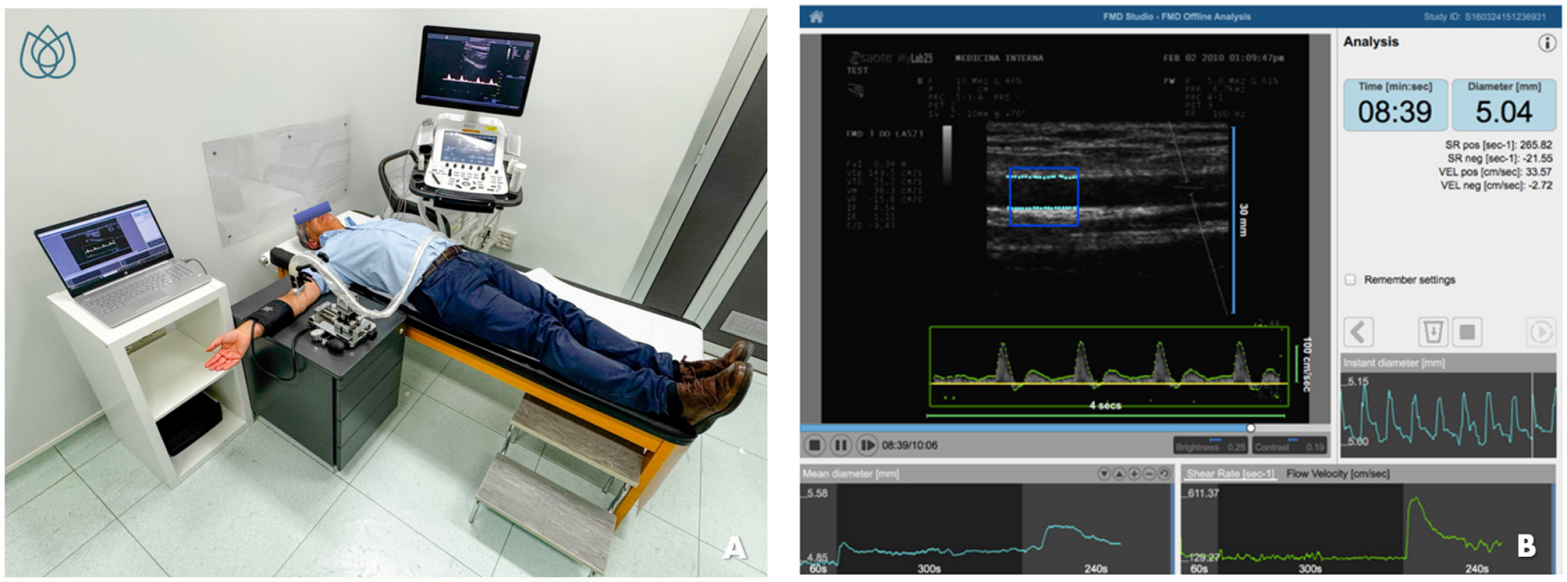
Evaluation of flow-mediated dilation (FMD) using FDA-approved software for edge detection, wall tracking, and shear-rate monitoring. **(A)** shows the patient's positioning and the setup of the equipment during the procedure. **(B)** illustrates the simple and intuitive interface of the software for semiautomatic FMD assessment. **(B)** is reproduced with permission from Quipu SRL, Pisa, Italy.

Flow-mediated dilation (FMD) refers to the increase in blood flow-induced tangential wall shear stress following a post-ischemic dilatory stimulus, a process that is dependent on NO release (45). This ultrasound-based and non-invasive technique is typically applied to a large-conductance vessel, such as the brachial artery. The ischemic stimulus is induced by inflating a pneumatic cuff on the forearm to supra-systolic pressure for 5 min. Upon cuff deflation, the sudden increase in brachial artery blood flow generates elevated shear stress, triggering NO release and subsequent vasodilation (45). Current guidelines recommend specific pre-test conditions, including fasting for 10–12 h, abstinence from smoking, vasoactive substances, alcohol, coffee, and tea, avoidance of physical activity, and maintaining an environmental temperature of 21–23°C under soft lighting (45). Despite consistent attempts in standardizing the procedure (46), the main limitation of this method is its high operator-dependency as well as a broad inter-subject variability (38). Recently, however, semi-automated software cleared by the Food and Drug Administration (FDA), such as Cardiovascular Suite^®^ (FMD Studio, QUIPU Srl, Pisa, Italy), has been introduced to enhance test reproducibility (Figure 2).

Peripheral arterial tonometry (PAT)-based technology (EndoPAT; Itamar Medical, Caesarea, Israel) has also emerged as an alternative for studying endothelial function (47). This method employs two plethysmographic sensors placed on the fingertips to detect pulsatile blood volume changes. An inflatable cuff is placed on one upper arm (study arm), while the contralateral arm serves as a control (48). The technique evaluates pulse wave amplitude (PWA) both at baseline and during wall shear stress. The reactive hyperaemia index (RHI) is calculated as the ratio of post-ischemic to baseline PWA using dedicated software (49). Compared to other non-invasive methods, PAT is simpler and more reproducible (48). RHI is considered an indicator of endothelial function and microvascular reactivity and is currently recognized as a key index for evaluating patients at high cardiovascular risk (50).

A recent systematic review of clinical methods for assessing endothelial function in COPD revealed that VOP, while historically significant, is now rarely used. Conversely, FMD remains the most widely applied technique in this context despite the limitations discussed above (51). PAT, while simpler and operator-independent, is however more expensive, and has been employed in relatively few studies (52). Furthermore, its utility in COPD might be influenced by the increased sympathetic tone commonly observed in these patients (51), and therefore, further investigation into its applicability and validation are warranted.

In summary, the clinical assessment tools discussed appear to be promising alternatives for the routine evaluation of ED in respiratory diseases, particularly COPD. However, the validation and standardization of these methods, the identification of reliable cut-off values, equipment-related costs (such as consumables in the case of PAT), and the need for adequately trained operators continue to hinder their widespread adoption.

### Evidence from clinical research

Clinical methods for assessing endothelial function have been employed in both stable COPD and AECOPD. A systematic review with meta-analysis, including 8 observational studies on 334 patients affected by COPD, concluded that ED studied by FMD is more pronounced in these patients compared to controls, with a statistically significant mean difference of −3.15% (95% confidence interval: −4.89, −1.40; *P* < 0.001). Additionally, ED was found to be independent from classical risk factors and cigarette smoking among COPD patients, thus underscoring a possible different intrinsic pathogenetic mechanism (53). This finding aligns with the conclusions of a previous systematic review and is supported by similar meta-analytical data, which identified a statistically significant mean difference in FMD values of −3.22% (95% confidence interval: −4.74, −1.69; *P* < 0.001), regardless of smoking habit (54, 55). Overall, this suggests that, while cigarette smoking is associated with endothelial damage through the triggering of the inflammatory cascade, the release of ROS and the reduction of NO bioavailability, this factor does not completely justify the increased cardiovascular risk in COPD (51). The relationship between ED and airflow limitation in COPD patients has been investigated in several studies. In particular, Eickhoff et al. observed a reduction of FMD values in relation to the increase of circulating inflammatory biomarkers (e.g., CRP, fibrinogen, interleukin-6) in patients affected by stable COPD, thus underlining a significant and dangerous association between airway obstruction, inflammation, ED and, therefore, cardiovascular risk (56). The relationship between lower values of FMD and a more impaired respiratory function was also explored in another study, further contributing to corroborate the close link between symptomatic worsening and lung function decline in moderate-to-severe COPD and impairment in endothelial function (57). Currently, only a few authors have investigated endothelial function using PAT-based technology in patients with COPD and this pathological condition is certainly associated with reduced RHI values (52, 55). However, the implications of this body of evidence and a comparison with FMD or other techniques are still scarce and should be explored with appropriately designed trials. Alongside stable COPD, endothelial damage analyzed with either FMD or PAT has also been studied in AECOPD. In this regard, a recent meta-analysis conducted on 5 studies, including a total of 279 patients, has contributed to shed light on this still poorly explored topic (58). FMD appeared to be significantly reduced in AECOPD especially due to the reduced bioavailability of NO. Two studies (24 total AECOPD) of the 5 analyzed in this meta-analysis used PAT as a technique for evaluating endothelial damage: in one study, only 50% of patients presented a reduced RHI (RHI < 0.40) (59), while in the other no relationship between a reduction in RHI values and AECOPD was found (60). Some authors have highlighted a significant endothelial function impairment during severe AECOPD, which tended to improve after the acute phase. This acute worsening and its subsequent reversal might be due to the temporary flare-up in systemic inflammation which involves the endothelium, leading to FMD alterations. This interesting evidence contributes to justify the observed increase in cardiovascular morbidity and mortality during AECOPD (61).

In conclusion, the aforementioned findings suggest that ED may not simply be an epiphenomenon arising from shared risk factors between COPD and CVD, but rather an intrinsic feature of COPD that could play a significant role in COPD-associated cardiopulmonary risk.

## Therapeutic advances: targeted therapies and rehabilitation

Although no specific medication for the treatment of isolated ED is currently approved, its amelioration still constitutes an unexpected but highly positive “side effect” of several commercially available drugs (32), including some antioxidant and nutraceutical preparations (9). Nonetheless, non-pharmacological therapies, and in particular pulmonary rehabilitation (PR) may play an important role in the improvement of ED among COPD patients.

### Molecular and pharmacological therapies

Traditional cardiovascular drugs, including renin-angiotensin pathway inhibitors and statins, may have benefits in COPD-related ED (62). In particular, statins, such as fluvastatin and atorvastatin, have demonstrated a reduction in mortality risk, decreased CRP levels, and a substantial improvement in endothelial function among COPD patients, as shown in the RODEO trial (NCT00929734), which highlighted endothelial improvements through FMD (63, 64). The effect of statins on ED might be related to the activation of anti-inflammatory pathways and conversely reduced CRP levels (64). Considering the role of platelet activation in ED, antiplatelet drugs may reduce ED and thrombotic risk in COPD. The PLATO trial (NCT00391872) found that ticagrelor not only reduces cardiovascular risk but also lowers bleeding risk compared to clopidogrel in COPD (65). These findings are supported by meta-analyses showing reduced mortality in COPD patients treated with antiplatelet agents (66). The antioxidant properties of N-acetylcysteine (NAC) have been investigated in clinical studies; however, its efficacy in managing ED in COPD remains controversial (67). Ongoing studies on antioxidant enzymes, including glutathione peroxidase and superoxide dismutase, have shown promising results on ED in animal models (68). Additionally, Ginkgo biloba extract (EGb) has demonstrated protective effects on pulmonary endothelial cells *in vitro* by upregulating the nuclear factor erythroid 2-related factor 2 (Nrf2) pathway (69).

The phosphodiesterase 4 (PDE4) inhibitor roflumilast, approved by FDA for COPD, inhibits leukocyte-endothelial cell interactions and is being evaluated in clinical trials for its anti-inflammatory properties through increased cAMP levels in inflammatory cells. Preliminary findings suggest it reduces exacerbation frequency and improves residual volume without significant changes in forced expiratory volume in 1 s (FEV_1_) (70, 71).

Finally, bosentan, an endothelin receptor antagonist, has shown potential benefits in hemodynamic parameters for COPD patients with pulmonary hypertension (PH) in experimental studies, though improvements in respiratory function were not observed (72, 73).

When considering potential pharmacological therapies, however, attention should be given to the variability in patient responses and their subsequent tolerability (74). In fact, the aforementioned drugs often present with side effects, so the risk/benefit ratio in the specific context of COPD should be carefully evaluated. Further research is needed to determine whether administering such drugs in the presence of a confirmed diagnosis of ED in COPD patients could provide an advantageous option for these patients.

### Pulmonary rehabilitation

Multidisciplinary pulmonary rehabilitation is a cornerstone of COPD management, as emphasized in international guidelines (75). Such programs improve morbidity, mortality, quality of life, and disability levels in COPD patients (76). Beyond pulmonary benefits, recent studies suggest that cardiac and pulmonary rehabilitation significantly enhance vascular health and reduce cardiovascular risk.

Regular physical exercise promotes endothelial repair by stimulating the mobilization of EPCs, enhancing endothelial nitric oxide synthase (eNOS) phosphorylation, and upregulating the activity of superoxide dismutase (32). EPC mobilization is modulated by factors such as NO bioavailability, interleukin-6 levels, and the presence of growth factors like vascular endothelial growth factor (VEGF) (77). Increased EPC levels have been shown to correlate with improvements in FMD, reductions in arterial stiffness, and a decrease in systemic inflammatory markers (78, 79).

Physical exercise has been shown to increase wall shear stress, leading to improved vascular function and structure (80). Studies on endothelial function in COPD patients undergoing pulmonary rehabilitation have reported improvements in arterial stiffness and cardiovascular parameters, including blood pressure and pulse (81). For example, an 8-week supervised training program enhanced FMD and endothelial function, even in small sample studies (82). Another study found that combining pulmonary rehabilitation with nitrate therapy produced superior endothelial outcomes compared to rehabilitation alone, likely due to insufficient training intensity to optimize wall shear stress (83).

Ambrosino et al. (84) recently conducted a longitudinal cohort study including 40 severe COPD patients and demonstrated a significant improvement (mean variation: + 1.62% ± 1.59) in endothelial function, assessed by FMD, after a personalized 4-week rehabilitation program. During the inpatient rehabilitation course, changes in lung function and spirometry parameters, as reflected by FEV_1_, were considered strong predictors of changes in FMD, thus confirming the direct association between the severity of airway obstruction and endothelial dysfunction. Improvements in endothelial function were observed also when stratifying patients according to demographic factors, treatment, and concomitant medical conditions; the only factor associated to a lower improvement of FMD values was hypercholesterolemia. The Authors therefore concluded that multidisciplinary rehabilitation may be able to improve endothelial dysfunction in COPD patients, therefore improving their cardiovascular risk profile.

Furthermore, a retrospective *post-hoc* analysis of 46 COPD patients undergoing a 5-week rehabilitation program found no significant impact of inhaled corticosteroids (ICSs) on endothelial function, suggesting an independent positive effects of rehabilitation on vascular health (85).

The importance of the above discussed data becomes undoubtedly clear when considering that, on the basis of meta-analytical evidence, for each percentage point reduction in FMD values, there is an increase of 12% in the risk of ischemic events (86). Therefore, given that pulmonary rehabilitation appears to positively affect FMD values, it could be hypothesized that PR may be identified as the most appropriate healthcare setting for the management of cardiovascular comorbidity in severe COPD patients.

In summary, the available evidence suggests that pulmonary rehabilitation may be a highly effective treatment strategy for addressing ED in COPD patients. Unfortunately, several barriers continue to limit regular access to pulmonary rehabilitation programs (87), including patients' misperception of rehabilitation as an additional burden rather than an opportunity, insufficient collaboration between primary care physicians and rehabilitation facilities, and a lack of caregiver support.

## Conclusions

COPD's systemic effects and cardiovascular risk are tightly linked through ED. Mounting evidence has shown that ED is a determinant feature of COPD and its improvement through different strategies may be linked to functional and clinical amelioration. Targeting ED early in COPD could potentially slow down disease progression and mitigate cardiovascular complications. Lifestyle modifications, pharmacological therapies, and rehabilitation programs provide complementary strategies to enhance endothelial health. Meanwhile, non-invasive techniques such as FMD and PAT, alongside emerging biomarkers, show promise for improving diagnosis and enabling personalized treatment. Despite a promising body of evidence, a strong effort is required in order to bring the study of ED within the current management of COPD patients, while several questions still remain unanswered. In fact, the optimal timing for initiating pulmonary rehabilitation and the most appropriate training regimen for COPD patients to reduce ED remain to be determined. Additionally, the potential synergistic effects of pulmonary rehabilitation combined with pharmacological or nutraceutical strategies (88) or the enhancement of its benefits through the administration of oxygen via high-flow devices (89) have not yet been fully explored. Future well-designed trials are needed to address these questions, as well as to identify the most suitable assessment tools for both research and clinical applications.

Referring COPD patients to intensive multidisciplinary pulmonary rehabilitation programs currently appears to be a practical and effective strategy for managing ED and, in turn, reducing their cardiopulmonary risk. Ultimately, exploring the role of ED in COPD's systemic manifestations remains a critical priority in both research and clinical practice.
